# Cohabitation duration, obstetric, behavioral and nutritional factors predict preeclampsia among nulliparous women in West Amhara Zones of Ethiopia: Age matched case control study

**DOI:** 10.1371/journal.pone.0228127

**Published:** 2020-01-27

**Authors:** Maru Mekie, Wubegzier Mekonnen, Meselech Assegid

**Affiliations:** 1 Department of Midwifery, College of Health Sciences, Debre Tabor University, Debre Tabor, Ethiopia; 2 School of Public health, College of Health Science, Addis Ababa University, Addis Ababa, Ethiopia; Ordu University, TURKEY

## Abstract

**Background:**

Preeclampsia is a major cause of maternal and perinatal mortality in developing countries. Identifying its risk factors is essential for early diagnosis and management. However, there has been a paucity of information on predictors of preeclampsia among nulliparous women in a resource limited setting. This study bridges the gap in this regard by examining the association of cohabitation duration, obstetric, behavioral and nutrition factors with preeclampsia among nulliparous women in West Amhara Zones of Ethiopia.

**Methods:**

Age matched case-control study design was employed among 110 preeclamptic and 220 non-preeclamptic women who came for delivery services at Felege Hiwot, Addis Alem, and Debre Tabor hospitals. Double population proportion formula with an assumption of 95% confidence interval, 80% power and a 2:1 control to case ratio was used to calculate sample size. Epi data 3.1 and SPSS 20 were used for data entry and analysis, respectively. Magnitudes of cohabitation duration, obstetric, behavioral and nutritional factors among nulliparous women with preeclampsia and their controls were calculated and the differences were tested with a Chi-square test. Conditional bivariable and multivariable logistic regression analysis were fitted to identify predictors of preeclampsia. Odds ratio along with their 95% confidence interval were used to identify the strength, direction and significance of association. Ethical clearance was secured from the research ethics committee of the School of Public Health in Addis Ababa University.

**Results:**

A total of 107 cases and 214 controls completed the interview giving a response rate of 97.27% for both cases and controls. Short cohabitation duration (AOR = 2.13, 95% CI (1.10, 4.1)), unplanned pregnancy (AOR = 2.35, 95% CI (1.01, 5.52)), and high body weight (AOR = 2.00, 95% CI (1.10, 3.63)) were found to be significant risk factors for preeclampsia. Whereas, antenatal advice about nutrition (AOR = 0.52, 95% CI (0.29, 0.96)), vegetable intake (AOR = 0.42, 95% CI (0.22, 0.82)) and fruit intake during pregnancy (AOR = 0.45, 95% CI (0.24, 0.87)) were protective factors for preeclampsia.

**Conclusion:**

Special attention should be given to nulliparous women with short cohabitation duration, unplanned pregnancy, and high body weight to minimize the effect of preeclampsia. Nutritional counseling shall be stressed during antenatal care follow ups.

## Introduction

Preeclampsia (PE) is a type of hypertensive disorder during pregnancy [1] which is defined as blood pressure (BP) ≥ 140/90 millimeter mercury (mmHg) measured on two occasions at least 4 hours apart or BP ≥ 160/110 mmHg on a single measurement with proteinuria diagnosed after 20 weeks of pregnancy in previously normotensive women [2]. PE is one of the top five causes of maternal morbidity and mortality [3, 4] which complicates 3% - 8% of all pregnancies worldwide [3, 5]. It is directly responsible for 70,000 maternal deaths annually at global level [6]. More than 16% of direct maternal deaths in Ethiopia are attributed to PE [7]. Studies indicated that fetal growth restriction, preterm birth, low birth weight, increased admission to neonatal intensive care unit, and low mean APGAR score were common complications among babies of women with PE [8, 9].

Though PE is the leading causes of maternal and perinatal mortality, the pathologic mechanism of the disease is not clearly understood. However, immunologic maladaptation of maternal antibodies to fetal and placental antigens might be assumed to cause inflammation which leads to abnormal placentation and placental hypoxia. Placental hypoxia is thought to lead to increased vascular sensitivity to angiotensin II and decrease the formation of vasodilators such as nitric oxide [2, 6, 10]. Women who have had a new partner and those with short duration of exposure to paternal antigen have a high risk of developing PE [11–13] despite there are inconsistent findings in this regard.

Preventive measures such as expansion of health facilities, construction of maternity waiting homes, training and deployment of health professionals were actions undertaken by the government of Ethiopia to reduce pregnancy related morbidity and mortality [14]. Regardless of the progress made, maternal mortality related to PE is still on the increase unlike that of abortion and other direct obstetrics causes of maternal mortality [4, 15, 16]. PE is a multi-factorial disease which is caused by socio-demographic factors, medical, obstetric, behavioral and nutrition related factors [12, 17, 18]. According to the WHO secondary data analysis, women who had chronic hypertension (HTN), gestational diabetes mellitus, low educational attainment, high body mass index, nulliparity, severe anemia, renal disease, lack of antenatal care (ANC), and urinary tract infection (UTI) were found to develop PE [18] at higher rate than healthy women. In the same manner, menarche at early age, non-fruit and vegetable users were found to be at risk to develop PE [12, 19, 20]. The risk of having PE in a subsequent pregnancy is also found to be dependent on the history of the disease in the first pregnancy [21, 22].

Though various studies have been conducted to determine risk factors for PE, those studies did not come up with conclusive findings on the effect of short cohabitation duration [12, 13, 23, 24], previous abortion [11, 25] and other obstetrics and behavioral factors on PE [19, 23]. In addition to the existing controversies, to the best of the authors’ knowledge; no study has been conducted about the effect of cohabitation duration on PE including other risk factors such as obstetrics, behavioral, and nutritional factors among nulliparous women in Ethiopia. Hence, this study aims to bridge the gap by identifying the effect of cohabitation duration, obstetric, behavioral, and nutritional factor on PE among nulliparous women in West Amhara Zones of Ethiopia.

## Methods and materials

### Study design and period

A facility based age matched case control study was conducted in three hospitals of West Amhara Zones from January 24 to April 24, 2018. Felege Hiwot (FHH) and Addis Alem (AAH) in Bahir Dar City, and Debre Tabor hospital (DTH) in Debre Tabor Town were selected due to their highest number of annual deliveries in West Amhara Zones.

### Study population

All nulliparous pregnant women with and without PE coming for delivery services at the three hospitals were part of the study population. Cases were nulliparous women who had BP ≥ 140/90 mmHg on two separate readings taken at least four hours apart or a single BP measurement of ≥ 160/110 mmHg and a proteinuria ≥ 300 mg per 24-hour urine collection or a dipstick reading of 1+ occurred after 20 weeks of gestation among previously normotensive women [2]. Controls were age matched nulliparous women with no PE. Nulliparous women with previous HTN history and those women who were severely ill were excluded in the study.

### Sample size determination and sampling procedures

Epi-Info version 7 was used to calculate the sample size at a 95% confidence interval, 80% power, and 2:1 control to case ratio. Proportions of short cohabitation duration of 45.6% among cases and 29.2% among controls were considered to determine the sample size [23]. Short cohabitation duration was selected for sample size calculations since it gave the largest possible sample among the variables. The final sample size after adding 10% non-response rate was 330 (110 cases and 220 controls).

The sample of 330 was proportionally allocated to the three hospitals based on the number of deliveries reported in those facilities in the year prior to the study (FHH = 5662, AAH = 1650 and DTH = 3200 deliveries). Hence, the allocated sample was 177, 51 and 102 for Felege Hiwot, Addis Alem and Debre Tabor Hospitals respectively. Since PE is a rare disease, all cases fulfilling the inclusion criteria were included by employing consecutive sampling techniques. For each PE cases, two age matched controls were selected. The selection of cases was made after PE was confirmed by a physician. Controls were age-matched normotensive nulliparous women who came for delivery services at the three hospitals. The controls were selected from the same hospitals as the cases to control the influence of context variation.

### Variables and measurements

With regards to measurement, PE is defined as increased BP with protein urea occurring after 20 weeks of pregnancy, irrespective of severity of the disease, in previously normotensive women [2]. Cohabitation duration is defined as the period of a sexual relationship with a partner before conception. This was measured by using the last normal menstrual period as a reference and counting back to the time of sexual initiation /marriage [26]. In this study, cohabitation duration was dichotomized as short (sexual cohabitation duration of < 12 months) or long (a sexual cohabitation duration of ≥ 12 months) with the biological father [12]. Likewise, obstetric factors including age at menarche, previous abortion, antenatal care, multiple pregnancy, iron intake, family history of PE, and family planning methods were considered. Information about obstetric factors were collected through an interview using a questionnaire. In addition, the patient’s medical records were reviewed in order to confirm the information collected in the interview.

The behavioral and nutritional factors included smoking, physical exercise, body weight measured by Mid-Upper Arm Circumference (MUAC), alcohol consumption, coffee consumption, and fruit and vegetable consumption. Information about behavioral and nutritional characteristics were collected during the interview using the same questionnaire. A minimum of at least one serving per week was considered as vegetable and fruit consumption in our study. MUAC was used to measure obesity /overweight instead of body mass index because MUAC is more accurate at indicating actual body fat and it remains consistent during pregnancy [27]. The measurement was taken, using a standard tape measure, midway between the olecranon of the elbow and the acromion process of the shoulder of the non-dominant arm. A MUAC cutoff point of ≥ 25 cm was used to define obesity in our study [28]. Data about socio-demographic factors such as maternal age, marital status, place of residence, economic status, level of education, occupation and medical factors (including HTN, anemia, UTIs, and diabetes mellitus) were captured during the interview.

### Data collection procedures and quality assurance

A structured, pretested questionnaire, adapted from the Ethiopian demographic and health survey (EDHS), was used to collect the socio-demographic, obstetric, behavioral, and nutritional information of the study participants. This standardized questionnaire was prepared in English and translated into Amharic and then translated back to English to check the consistency and quality of the translation. A document review was conducted to confirm the diagnosis of PE and the previous medical history using a prepared checklist. MUAC was used to assess obesity and the measurement was taken at the end of the interview session. Data collection was conducted by five BSc midwives and supervised by 2 MSc clinical midwives. To minimize measurement bias, two days of training on the questionnaire and interview techniques was given to the data collectors and supervisors. Pretesting was conducted on 5% of the study sample and modifications were made to the study tools to ensure quality.

### Data management and analysis

The collected data were entered in a template created on Epi data version 3.1. The template integrated an internal consistency check in order to minimize data entry errors. After cleaning the data, the data were exported to SPSS version 20 for analysis. Frequency distribution was run for important variables and recoding was done Descriptive statistics were computed to see the difference in the prevalence of PE across cases and controls and a Chi-square test was used to assess the level of significant difference. Since we have used the matched case control study design, matched analysis (conditional logistic regression analysis) was used to control bias. Thus, variables which were found to be associated with PE in the chi-square test were included in the conditional binary logistic regression. Furthermore, different variables including cohabitation duration, obstetric, behavioral, and nutritional factors were included in the conditional multivariable logistic regression model to single out the effect of each covariate with PE. Multi-collinearity between independent variables was checked. The Hosmer-Lemeshow goodness of fit test was used to assess the fitness of the model. The model was fit at X^2^ = 10.05 and p-value of 0.26. Both crude and adjusted odds ratio (OR) along with their 95% confidence intervals were used to measure the significance, strength, and direction of association of PE with its predictor variables.

### Ethical consideration

Ethical clearance was secured from the research ethics committee (REC) of the College of Health Sciences in Addis Ababa University. Written consent was obtained from each study participant after a detailed description of the aim and procedures used in the study were explained. For participants who were under 16, oral consent from their parents/guardians was also required. This was done after the parents were explained the purpose of the study, and written assent was taken from the participants. The privacy of respondents and confidentiality of information was ensured.

## Results

### Sociodemographic characteristics of the study participants

A total of 321 nulliparous women with PE and their age matched controls (107 cases and 214 controls) completed the interview giving a response rate of 97.27% for both cases and controls. Nine participants (3 cases / 6 controls were excluded in the study due to the failure to get age matched controls). Age was the matching variable in our study and a five year age group was used as the matching criteria. The mean age of the study participants was 22.92 ± 4.64 SD years and the median age was 23.0 years. The proportion of nulliparous women in the age groups 15–19, 20–24, 25–29, 30–34 years were 31.3%, 29.4%, 30.0% and 9.3% respectively.

A higher proportion of cases, 55 (51.4%), than controls, 36 (16.8%), were found to have sexual cohabitation of less than 12 months. The median cohabitation duration among cases and controls were found to be 11 (IQR = 18) and 20 (IQR = 12.25) months, respectively. On the other hand, the median monthly household income of the study participants was 3000 ETB with a minimum and maximum of 600 and 12,000 ETB respectively. More than half of the cases, 60 (56.1%), and 98 (45.8%) controls had a monthly household income of less than 3000 ETB. The distribution of nulliparous women did not have a significant difference except for cohabitation duration and attended levels of education (Table 1).

**Table 1:** Distribution of case and control nulliparous women, 2018.

Variable	Cases Number (%)	Controls Number (%)	Chi-square	P- value
**Residence: (N = 321)**				
Urban	63 (58.88)	148 (69.16)	3.347	0.067
Rural	44 (41.12)	66 (30.84)		
**Marital status: (N = 321)**				
In union	94 (87.85)	200 (93.46)	2.912	0.088
Not in union	13 (12.15)	14 (6.54)		
**Age at marriage:(N = 321)**				
< 20 years old	57 (53.27)	94 (43.93)	2.501	0.114
≥20 years old	50 (46.73)	120 (56.07)		
**Cohabitation: (N = 321)**				
<12 months	55 (51.40)	36 (16.82)	41.992	< 0.001
≥ 12 months	52 (48.6)	178 (83.18)		
**Education status: (N = 321)**				
No education	45 (42.06)	46 (21.50)	16.330	0.001
Primary/informal	30 (28.04)	69 (32.24)		
Secondary	15 (14.02)	55 (25.70)		
Higher Education	17 (15.89)	44 (20.56)		
**Main Occupation: (N = 321)**				
House maker	27 (25.23)	52 (24.30)	9.902	0.078
Student	5 (4.67)	8 (3.74)		
Private business	16 (14.95)	56 (26.17)		
Farmer	39 (36.45)	49 (22.90)		
Hand craft work	5 (4.67)	9 (4.20)		
Employed	15 (14.02)	40 (18.69)		
**Monthly income: (N = 321)**				
<3000 ETB	60 (56.07)	98 (45.79)	3.016	0.082
≥3000ETB	47 (43.93)	116 (54.21)		

### Factors associated with PE among nulliparous women

Table 2 (below) revealed the predictors of PE among nulliparous women in West Amhara Zones of Ethiopia. The odds of developing PE were found to be 2.13 times higher among nulliparous women who had a cohabitation duration of less than 12 months (AOR = 2.13, 95% CI (1.10, 4.11)) compared with those with a cohabitation duration of 12 months or greater. Similarly, the risk of experiencing PE was found to be 2.35 times higher among women who had unplanned pregnancy (AOR = 2.35, 95% CI (1.01, 5.52)) compared with their counterparts. On the other hand, the risk of facing PE was 48% lower among nulliparous women who received nutritional counseling during ANC follow-up compared with those who did not receive counseling (AOR = 0.52, 95% CI (0.29, 0.96)). Moreover, women who had frequent ANC visits had a low risk of experiencing PE compared with their counterparts (COR = 0.51, 95% CI (0.29, 0.92)) though the significance of the association vanished when controlling other confounding factors. Similarly, nulliparous women who had multiple pregnancy (COR = 1.97, 95% CI (1.03, 3.78)) and users of barrier methods of contraception (COR = 1.82, 95% CI (1.00, 3.33)) had higher risk of developing PE compared with their counterparts, although the significance vanished when controlling confounding factors.

**Table 2:** Conditional multivariable analysis of risk factors on preeclampsia among nulliparous women, 2018.

Variables	Cases	Controls	COR:95% CI	AOR:95% CI
**Cohabitation duration: (N = 321)**				
<12months	55 (51.4)	36 (16.8)	2.80 (1.90, 4.13)	2.13 (1.10, 4.11)*
≥ 12 months	52 (48.6)	178 (83.2)	1	1
**Education status: (N = 321)**				
No education	45 (42.1)	46 (21.5)	1.84 (1.03, 3.29)	1.05 (0.50, 2.23)
Primary/informal	30 (28.0)	69 (32.2)	1.11 (0.60, 2.05)	1.02 (0.38, 2.74)
Secondary	15 (14.0)	55 (25.7)	0.79 (0.39, 1.60)	1.31 (0.48, 3.56)
Higher Education.	17 (15.9)	44 (20.6)	1	1
**Planned pregnancy: (N = 321)**				
Yes by then	65 (60.7)	169 (79.0)	1	1
Yes latter	10 (9.3)	24 (11.2)	1.13 (0.57, 2.24)	1.56 (0.56, 4.39)
No	32 (29.9)	21 (9.8)	2.31 (1.48, 3.60)	2.35 (1.01, 5.52)*
**Number of ANC visits:(N = 260)**				
1–3 visits	57 (79.2)	119 (63.3)	1	1
≥4 visits	15 (20.8)	69 (36.7)	0.51 (0.29, 0.92)	0.59 (0.26, 1.31)
**Nutritional counseling at ANC: (N = 260)**				
Yes	44 (61.1)	163 (86.7)	0.45 (0.27, 0.73)	0.52 (0.29, 0.96)*
No	28 (38.9)	25 (13.3)	1	1
**Multiple pregnancy: (N = 321)**				
Yes	9 (8.4)	5 (2.3)	2.02 (1.02, 4.01)	1.11 (0.36, 3.39)
No	98 (91.6)	209 (97.7)	1	1
**Family planning:(N = 321)**				
Non users	41 (38.3)	78 (36.4)	1	1
Hormonal users	50 (46.7)	126 (58.9)	0.82 (0.53, 1.26)	0.74 (0.35, 1.58)
Barrier users	16 (15.0)	10 (4.7)	1.82 (1.00, 3.33)	1.34 (0.41, 3.15)
**Alcohol use: (N = 321)**				
Yes	47 (43.9)	58 (27.1)	1.65 (1.12, 2.43)	1.29 (0.72, 2.34)
No	60 (56.1)	156 (72.9)	1	1
**Coffee per day: (N = 247)**				
≤ 4 cups	71(80.7)	149 (93.7)	1	1
> 4 cups	17 (19.3)	10 (6.3)	1.96 (1,14, 3.37)	1.67 (0.69, 4.05)
**Vegetable consumption:(N = 321)**				
Yes	73 (68.2)	184 (86.0)	0.53 (0.35, 0.80)	0.42 (0.22, 0.82)*
No	34 (31.8)	30 (14.0)	1	1
**Fruit consumption: (N = 321)**				
Yes	50 (46.7)	138 (64.5)	0.62 (0.42, 0.91)	0.45 (0.24, 0.87)*
No	57 (53.3)	76 (35.5)	1	1
**MUAC: (N = 321)**				
< 25 cm	55 (51.4)	191 (89.3)	1	1
≥ 25cm	52 (48.6)	23 (10.7)	3.10 (2.12, 4.53)	2.00 (1.10, 3.63)*
**Family history of HTN: (N = 321)**				
Yes	20 (18.7)	7 (3.3)	2.18 (1.29, 3.68)	2.10 (0.96, 4.61)
No	87 (81.3)	207 (96.7)	1	1
**UTI: (N = 321)**				
Yes	18 (16.8)	10 (4.7)	2.12 (1.28, 3.52)	1.34 (0.40, 3.25)
No	89 (83.2)	204 (95.3)	1	1

* Significant at P< 0.05 in multivariable model, 1 = reference, COR: crude odds ratio, AOR: adjusted odds ratio

With regards to behavioral and nutritional factors, alcohol abuse, drinking more than 4 cups of coffee per day, and fruit and vegetable consumption were found to be associated with PE. Alcohol abuse and taking more than 4 cups of coffee per day were found to be significantly associated with the risk of acquiring PE in the crude analysis (COR = 1.65, 95% CI (1.12, 2.43) and (COR = 1.96, 95% CI (1.14, 3.37) respectively, however, the significance of association did not persist when controlling confounding factors. The conditional multivariable logistic regression analysis revealed that nulliparous women who include fruits and vegetables in their diet had a 58% (AOR = 0.42, 95% CI (0.22, 0.82)) and a 55% (AOR = 0.45, 95% CI (0.24, 0.87)) reduction in experiencing PE compared with their counterparts. Similarly, the odds of acquiring PE were 2 times higher among women with an MUAC of 25 centimeters or more (AOR = 2.00, 95% CI (1.10, 3.63)) compared with those with an MUAC of less than 25cm.

A family history of hypertension (HTN) and urinary tract infection (UTI) were among the medical factors which were found to be significantly associated with PE at a bivariable level; (COR = 2.18, 95% CI (1.29, 3.68)) and (COR = 2.12, 95% CI (1.28, 3.52)) respectively. Nevertheless, the association vanished in both medical factors after adjusting for other confounders.

## Discussion

This study tried to show the association between cohabitation duration, obstetric, behavioral, and nutritional factors with PE among nulliparous women who came for delivery services in the selected hospitals of the West Amhara Zones. The following factors were all found to be significant predictors of PE in the conditional multivariable model; a short cohabitation duration, unplanned pregnancy, nutritional counseling during ANC visits, body weight, fruit, and vegetable consumption.

The odds of acquiring PE were 2.13 times higher among women with short cohabitation durations (AOR = 2.13, 95% CI (1.10, 4.11)) compared with their counterparts. The findings of this study is consistent with other studies in India [12] and Egypt [24]. The higher odds of acquiring PE among women who had a short cohabitation duration could be ascribed to lack of adaptation of maternal antibody to the paternal antigen [6, 29] which results in inflammation, abnormal placentation, and placental hypoxia. Women who had a long duration of cohabitation could have a chance to develop immunologic tolerance to the paternal antigen [13]. In addition to reducing the risk of PE, delaying pregnancy also allows the couple to become more financially secure and more psychologically prepared for the pregnancy. This ensures family stability and a reduced risk of developing PE. However, the finding of our study is not in agreement with other studies conducted in Nigeria [11] and South Africa [13] which found no significant relationship between a short cohabitation duration and PE. The justification for the discrepancy might be due to the difference in sample size (a small number of cases in Nigeria), the study population and study design (use of mixed parity in South Africa).

The women who received nutritional counseling during ANC follow-up had a 50% lower risk of acquiring PE compared with their counterparts in this study (AOR = 0.52, 95% CI (0.29, 0.96)). This is in line with a study conducted in select facilities in Addis Ababa which documented a lower risk of PE among women who receive nutritional counseling during ANC visits [22]. The possible justification might be that women who receive nutritional counseling are able to avoid an unhealthy diet which predisposes them to PE compared with those who did not get this nutritional advice.

Moreover, this study showed that the odds of experiencing PE were 2.35 times higher among women who had unplanned pregnancies (AOR = 2.35, 95% CI (1.01, 5.52)). The high risk of facing PE among women with unplanned pregnancy might be related to a lack of knowledge of the importance of proper healthcare and poor decision making. These women are usually young and inexperienced which may lead to poor decision making related to healthcare and an inability to decide when it is necessary to seek professional medical assistance. This finding in our study is not congruent with a study done in Addis Ababa [22]. This could be due to a variation in the study population as the aforementioned study was conducted among women with mixed parity in contrast to our study. Multiparous women may be better decision makers and have enough experience to understand delaying pregnancy is beneficial. Moreover, the contextual difference among study areas could also explain the variation. Accessibility to health facilities in Addis Ababa is different compared with the Amhara region. Encouraging women to have planned pregnancies can improve the health of the mother as well as the fetus by reducing the risk of post-partum hemorrhage, low birth weight, and preterm birth which are greatly related to PE. Reducing unplanned pregnancy is important for achieving the national new born and child survival strategy by reducing the risks of neonatal death [14].

Similar with previous studies [20, 30], consumption of fruits (AOR = 0.42, 95% CI (0.22, 0.82)) and vegetables (AOR = 0.45, 95% CI (0.24, 0.87)) during pregnancy were found to be protective factors for PE. Nulliparous women who reported that they included fruits and vegetables in their diet had more than a 50% lower risk of developing PE. Including fruits and vegetables in their daily diet during pregnancy has numerous benefits for the mother as well as the growing fetus. The existence of ample antioxidants, vitamins, minerals, and dietary fiber in fruits and vegetables might be one of the mechanisms for reducing the risks of PE [20]. Providing counseling on a healthy diet during pregnancy could be more effective than during other periods of life since most women are highly motivated to seek dietary advice during pregnancy. Moreover, the cost of dietary modification is low compared to medical interventions [31]. Likewise, this study indicated that the odds of developing PE were found to be 2 times higher among nulliparous women with heavy weight (AOR = 2.00, 95% CI (1.10, 3.63)). The possible explanation for how high body weight predisposes the women to PE could be related to increased pro-inflammatory factors which results in an exaggerated inflammatory response [32]. Our finding is supported by studies in Bahir Dar, Ethiopia [28] and New Delhi, India [17] which found a higher risk of acquiring PE among obese women.

This study has some limitations to be taken in to account. Due to the retrospective nature of the study design, our data may be subject to recall bias despite the fact that effort was made to minimize recall bias by reviewing the clients’ charts. Similarly, participants may not have disclosed their exact experiences related to sexual and behavioral characteristics due to cultural and social desirability bias. The study was done in a hospital setting which might not be generalizable to the general population despite our effort to incorporate three levels of hospitals (referral, general and primary) to increase its representation.

## Conclusion

Having a short cohabitation duration, unplanned pregnancy, and high body weight were found to be risk factors for PE while nutritional counseling during ANC visits, and fruit and vegetable consumption were preventive factors. Encouraging pregnant women to practice preventive factors is key to preventing PE. Similarly, the aforementioned risk factors should be considered as health education messages by health care providers to teach mothers to practice preventive measures. Health professional could also use these factors for early prediction, diagnosis, and management of PE and its complications.

## Supporting information

S1 Dataset (DOCX)

S2 Dataset (SAV)

## Abbreviations

AAH: Addis Alem Hospital
ANC: Antenatal Care
BP: blood pressure
DTH: Debre Tabor Hospital
ETB: Ethiopian Birr
FHH: Felege Hiwot Hospital
HTN: hypertension
IQR: Inter Quartile Range
mmHg: millimeter mercury
MUAC: mid upper arm circumference
PE: preeclampsia
UTI: urinary tract infection
