# Supplementary material for: Cohabitation duration, obstetric, behavioral and nutritional factors predict preeclampsia among nulliparous women in West Amhara Zones of Ethiopia: Age matched case control study
Source: PLoS One. 2020 Jan 27;15(1):e0228127. doi: 10.1371/journal.pone.0228127 (PMC6984729; doi:10.1371/journal.pone.0228127)
Supplement: S1 Dataset — (DOCX) [file pone.0228127.s001.docx]

**Supporting information for minimal dataset**

We have attached the minimal datasets used to draw conclusion for this study. Here are the description of variables for the attached datasets.

There are 22 variables in the datasets (these are variables used to draw conclusion not the whole data).

- **Variable 1: Q103 STAT**: is about the status of participates (coded as 1 for cases (preeclampsia) and 0 for controls (participants with no preeclampsia)).
- **Variable 2, Q104ID**: is the identification of study participants (1-321)
- **Variable 3, Q207COHA**: This variable is about duration of cohabitation of the study participants in months.
- **Variable 4, Q212OCUP**: This variable is about occupation of the study participants. The main occupation of the study participants reported were ( 1 house maker, 2 student, 3 private business, 4 farmer, 5 hand craft worker, and 6 government employee)
- **Variable 5, Q213 INCM:** This variable is about household income. The median value was 3000 Ethiopian Birr. Dichotomized as < 3000ETB and >= 3000 ETB.
- **Variable 6, Q302PPX:** This variable is about the status of pregnancy whether planned or not. The variable is categorized in to 3 as planned by then, planned but later and unplanned).
- **Variable 7, Q311ANC**: This variable is about antenatal care follow-up. Dichotomized as yes and no).
- **Variable 8, Q316NUTC**: This variable is about nutritional counseling during antenatal care follow-up. It is yes or No question.
- **Variable 9, Q319MPX**: This variable is about number of fetuses in the index pregnancy. Categorized as one fetus and more than one fetuses)
- **Variable 10, Q401DRNK**: This variable is about alcohol drink during the index pregnancy. Categorized as yes / no answer.
- **Variable 11, Q414VEGT**: This variable is about vegetable consumption during pregnancy. It is yes or no answer.
- **Variable 12, Q416 FRUT**: This variable is about fruit consumption during pregnancy. It is yes or no answer.
- **Variable 13, Q501FHTN**: This variable is about family history of hypertension. It is yes or no question.
- **Variable 14, Q507UTI**: This variable is about urinary tract infection of the study participants during the current pregnancy. It is a yes or no questions.
- **Variable 15, age group**: This variable is the categorized age of the study participants. Five year age group was used.
- **Variable 16 fake_time**: This is fake time set for analysis of matched case control study. Categorized as 1 and 0. This variable was derived from the status of participants for the sake of cox regression/ conditional logistic regression/.
- **Variable 17, COHA_R**: This variable is cohabitation duration of the study participants prior to conception in the recent pregnancy. It is dichotomized as short and long duration of cohabitation.
- **Variable 18, NCOF_R**: This variable is about numbers of coffee taken in cups during the recent pregnancy. Dichotomized as <= 4 cups per day and > 4 cups per day.
- **Variable 19, NANC_R**: This variable is about numbers of antenatal care visits in the recent pregnancy. Dichotomized as 1-3 visits and 4 and more.
- **Variable 20, MUAC_DI:** This variable is about MUAC of the study participants which as used to assess the effect of body weight on preeclampsia. For analysis purpose it was dichotomized as < 25cm and >= 25 cm.
- **Variable 21, LEDU_R**: This variable is about level of education of the study participants. The variable has 4 categories (1 no education, 2 informal/primary, 3 secondary and 4 higher education.)
- **Variable 22, FP_RECODED**: This variable is about use of family planning method. The variable has 3 options (1 non users, 2 hormonal method users, and 3 barrier method users.)
